# Retinal vascular density in children with hypertension

**DOI:** 10.1007/s00467-025-07076-7

**Published:** 2026-01-08

**Authors:** Katarzyna Maćkowiak-Lewandowicz, Anna Rzeszotarska, Marta Pawlak, Ewa Goździewska, Elżbieta Cymerys, Joanna Siwiec-Prościńska, Jacek Zachwieja, Anna Gotz-Więckowska, Danuta Ostalska-Nowicka

**Affiliations:** 1 Department of Pediatric Nephrology and Hypertension, Poznan University Od Medical Sciences, Poznań, Poland; 2 Department of Ophthalmology, Regional Hospital in Poznan, The Greater Poland Specialist Center, Poznań, Poland; 3 Department of Ophthalmology, Poznan University of Medical Sciences, Poznań, Poland

**Keywords:** Retina, Hypertension, Kidney injury, Children, Adolescents

## Abstract

**Background:**

Early detection of ophthalmological and kidney complications of hypertension in children and adolescents may play a significant role in prophylaxis and prevent irreversible organ damage. This study aimed to assess standard kidney injury markers (creatinine, urea, uric acid, cystatin C, 24-h microalbuminuria), as well as potential ophthalmological changes using optical coherence tomography (OCT) and optical coherence tomography angiography (OCT-A) in the early course of newly diagnosed hypertension in children and adolescents.

**Methods:**

The study group consisted of 56 children and adolescents with newly diagnosed hypertension who had not received antihypertensive treatment prior to the study. Fifteen individuals served as controls. The ECHO, abdominal ultrasound, ophthalmological examination, urine and blood tests were performed.

**Results:**

The concentration of cystatin C was increased in patients with hypertension. Children and adolescents with hypertension had decreased values of GFR (90.31 ± 13.00 ml/min/1.73 m^2^), estimated by the Filler equation, compared to subjects with optimal values of blood pressure (99.00 ± 9.27 ml/min/1.73 m^2^). The data revealed statistically significant differences in the retinal vessel density analyzed by OCT-A, which was decreased in the control group compared with the study group.

**Conclusions:**

Pediatric patients with newly diagnosed hypertension have increased concentrations of cystatin C and hypofiltration estimated by the Filler equation. OCT-A might be considered a diagnostic tool for better understanding the early process of microvascular changes and the influence of concomitant comorbidities in newly diagnosed systemic hypertension.

**Graphical abstract:**

**Figure:**
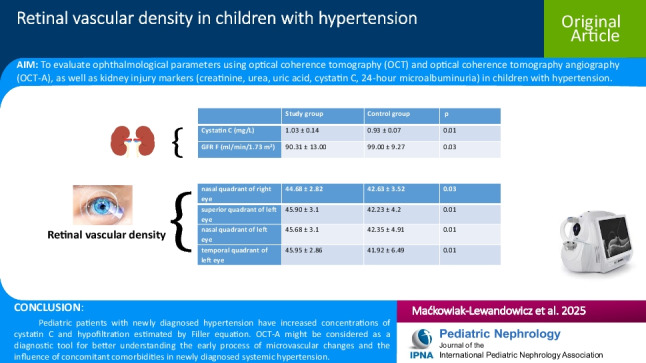
A higher-resolution version of the Graphical abstract is available as  Supplementary information

**Supplementary Information:**

The online version contains supplementary material available at 10.1007/s00467-025-07076-7.

## Introduction

Hypertension remains a significant public health concern with increasing prevalence among the paediatric population [1]. The increased incidence of overweight and obesity due to lifestyle changes during the COVID-19 pandemic, as well as the increasing survival rate of extremely premature neonates, are only a few of the well-known risk factors that may affect the cardiovascular system of paediatric patients [2, 3].

Hypertension, even in a very early stage, may affect both retinal and renal microvasculature. In the past, the detection of ophthalmological alterations was limited, as it relied only on fundoscopy. Optical coherence tomography angiography (OCT-A) is a non-invasive technique used for imaging the retinal capillary plexuses and choriocapillaris by tracing the red cell movement inside the vessels, thereby accurately depicting the vessels without using intravascular dyes [4]. Retinal microvascular changes, evaluated through OCT-A, provide important insights into systemic microvascular health. OCT-A was applied in the studies regarding the comparison of its parameters in adults with systemic hypertension and healthy controls. Adult patients with diagnosed hypertension had significantly lower superficial and deep vascular densities in the macula in comparison with healthy controls [5]. Limited data exist on OCT-A evaluation of retinal microvascular changes in newly diagnosed pediatric hypertension [6]. Also, the normal, physiological structure of the retina, choroid and optic nerve may be affected by the vascular changes. Optical coherence tomography (OCT) was used in the evaluation of choroidal thickness, as well as retinal nerve fibre layer (RNFL), macula and optic nerve head (ONH) in adult patients diagnosed with systemic hypertension [7–10]. Most of the studies were focused on ophthalmological evaluation in patients with long-lasting systemic hypertension, while the first objective side effects may occur in a very early stage and remain undetectable without proper diagnostic tools.

Elevated blood pressure has been linked to multiple nephrological adverse clinical outcomes, including renal insufficiency and proteinuria. Multiple mechanisms are involved in the determination of kidney glomerular, tubular and interstitial injuries in hypertension. Renal microcirculation is tightly regulated by tubuloglomerular feedback, which adjusts afferent arteriole tone in response to changes in renal perfusion pressure and sodium levels. Damage to preglomerular arteries and arterioles results in ischemia and the progressive narrowing of the preglomerular microcirculation. Microvascular remodeling and rarefaction reduce perfusion, leading to ischemia and tubular injury [11]. The proximal tubule, which has the highest mitochondrial content in the kidney and relies on oxidative phosphorylation to generate ATP, is vulnerable to ischemic insults and mitochondrial dysfunction. Histologically, pathological changes in the proximal tubules are more consistent than changes to the glomeruli or the loop of Henle in kidney injury [12]. There is a group of proximal tubule markers, which are analyzed in scientific research such as NGAL (neutrophil gelatinase associated lipocalin), KIM-1 (kidney injury molecule-1), megalin. In clinical routine tests cystatin C, β2-microglobulin, α1-microglobulin are used to assess tubular proteinuria; only cystatin C can be useful to recognize hyper- or hypofiltration (based on the value of GFR estimated by the Filler equation) [13].

The main aim of this study was to assess standard kidney injury markers (creatinine, urea, uric acid, cystatin C, 24-h microalbuminuria), as well as potential ophthalmological changes using optical coherence tomography (OCT) and optical coherence tomography angiography (OCT-A) in the early course of newly diagnosed hypertension in children and adolescents.

## Material and methods

The study group consisted of 56 patients with newly diagnosed hypertension (less than 6 months), who were hospitalized in the Department of Paediatric Nephrology and Hypertension at Poznań University of Medical Sciences between January 1, 2021, and December 31, 2023. All participants were treatment naïve. The study group was divided into subgroups: twenty-four patients with primary hypertension and thirty-two subjects with secondary hypertension. The exclusion criteria were as follows: (1) age < 6 years, (2) secondary hypertension connected with renal artery stenosis or kidney failure, (3) heart failure, (4) diabetes, (5) coexistence of neoplastic and/or any infectious diseases, (6) complications in the course of the perinatal period. In the group with secondary hypertension, 52% of subjects were diagnosed with kidney disease (mainly congenital abnormalities in the kidneys and urinary tract). Cardiovascular defects presented in 31% of patients, mainly aortic coarctation. The last 10% of patients presented a secondary hypertension phenotype (age < 10 years old, complications of hypertension stage 2) without a single dominant etiology. This group included children with severe, complicated hypertension, where the precise etiology may only be identified at an older age due to diagnostic limitations. The control group consisted of 15 age-matched healthy children and adolescents without hypertension. In both groups, less than half were boys (42.8% in the study group and 46.6% in the control group). Subjects enrolled in the control group had to meet the following inclusion criteria: (1) age 6–18 years, (2) no arterial hypertension, (3) normal clinical examination without a history of chronic diseases, (4) no clinical or laboratory signs of infection, (5) normal concentration of serum cortisol, glucose, thyroid hormones, electrolytes, (6) no aberrations in ECHO, (7) no complications in the course of the perinatal period. Although these patients were initially referred to the Nephrology Department as hypertension suspects, a thorough clinical evaluation allowed for ruling out hypertension and other chronic conditions. They did not meet criteria for white coat hypertension (which would require elevated office BP with normal ABPM), and all had normal ABPM values, laboratory tests, and cardiac ultrasound.

ECHO (S70N, GE) was performed in all patients from both study and control groups with the assessment of left ventricular hypertrophy marker (left ventricular mass index—LVMI > 95 centile), by a pediatric cardiologist as part of the patient’s evaluation during hospitalization. The left ventricular mass (LVM) was calculated using the formula of Devereux et al. [14], by the following equation:

$$\mathrm{LVM}\:=\:0.8\;\lbrack1.04\:\times\:{(\mathrm{interventricular}\;\mathrm{septal}\;\mathrm{thickness}\:+\:\mathrm{posterior}\;\mathrm{wall}\;\mathrm{thickness}\:+\:\mathrm{end}-\mathrm{diastolic}\;\mathrm{diameter})}^3\:-\:(\mathrm{end}-\mathrm{diastolic}\;\mathrm{diameter})^3\rbrack\:+\:0.6$$

The LVMI was calculated as LVM divided by the body surface area and measured by g/m^2^ [by the formula LVMI = LVM/height (m)^2.7^].

An abdominal ultrasound with Doppler test (H60, Samsung) was conducted by the radiologist to evaluate kidney arterial blood flow (the resistive index (RI) is a calculated flow parameter in ultrasound, derived from the maximum, minimum, and mean Doppler frequency shifts during a defined cardiac cycle). In our study every patient with hypertension had US as a routine test to exclude flow disorders in renal vessels.

The urine samples were collected on the same morning as the blood samples. Then concentrations of creatine, cystatin C, uric acid, urea, glucose, lipid profile in the blood samples and urine analysis, as well as microalbuminuria, concentration of sodium (analyzed from 24-h urine collection) were performed. Laboratory tests were performed using a standardized method, according to the manufacturer’s instructions.

Glomerular filtration rate was estimated by Filler and Schwartz equations. Filler’s formula is based on concentrations of serum cystatin C [15]:

$$\mathrm{Log}(\mathrm{eGFR})\hspace{0.17em}=\hspace{0.17em}1.962\hspace{0.17em}+\hspace{0.17em}[1.123\hspace{0.17em}\times \hspace{0.17em}\mathrm{log}(1/\mathrm{cystatin})].$$

The Schwartz equation is analyzed from concentrations of serum creatinine and individual height [16]:

$$\mathrm{eGFR}\hspace{0.17em}=\hspace{0.17em}(0.413\hspace{0.17em}\times \hspace{0.17em}\text{height in cm})/\text{serum creatinine}.$$

The day before hospitalization every patient had 24-h ambulatory blood pressure monitoring (ABPM) obtained from Mobil-O-Graph, IEM.

The ophthalmological examination was performed by one certified ophthalmology specialist in the Clinic of Ophthalmology under standard conditions, after prior preparation and proper hydration of the patient. Each patient underwent a complete ophthalmological evaluation including best corrected visual acuity (BCVA), cycloplegic refraction, intraocular pressure measurement (CT-80 computerized tonometer, Topcon, Japan), slit-lamp anterior segment assessment, indirect dilated fundus examination with Volk lens, as well as OCT and OCT-A (DRI-Triton Topcon SS-OCT, Japan).

The ophthalmological examination was performed between 8:00 and 11:00 a.m. Peripapillary RNFL (retinal nerve fibre layer) thickness parameters were automatically calculated by IMAGEnet software compatible with DRI-Triton SS-OCT. The average, inferior, superior, nasal, and temporal quadrant RNFL thicknesses were evaluated. For choroidal thickness evaluation, the macula radial 9 × 9 mm protocol was used. At the next step the choroidal thickness defined as the perpendicular distance between the retinal pigment epithelium/Bruch’s membrane layer and sclero-choroidal interface was measured manually in 7 locations (at the subfoveal region, at 500 um, 1000 um and 1500 um nasal to the fovea and at 500 um, 1000 um, 1500 um temporal to the fovea) by one examiner (A.R.) blinded to the diagnosis of the participants. OCT-A of the macula using 6 × 6 mm scans was also performed and the vessel density of the superficial capillary plexus (SCP) was measured automatically as the percentage of pixels occupied by blood flow in the central area and in four quadrants (inferior, superior, nasal, and temporal) that were used in statistical analysis.

Patients were classified as normotensive and hypertensive subjects according to 24-h ABPM. Hypertension was diagnosed based on the 2022 AHA guidelines by Flynn et al. and ABPM reference percentiles described by Wühl et al. [17, 18]. Hypertension was confirmed based on 24-h ABPM (Mobil-O-graph, IEM), when mean day and night systolic and/or diastolic blood pressure was at or above the 95th percentile for age, sex and height. For boys and girls aged 16 or older, to diagnose hypertension the mean values of day and night systolic and/or diastolic blood pressure were > 135/85 mmHg and > 120/70 mmHg [19]. Additionally, the mean arterial pressure (MAP) values of every subject were analyzed. The equation presented below was used to assess the MAP:

$$\textrm{MAP}=\text{diastolic blood pressure} +\mathrm{1/3}\times(\textrm{systolic blood pressure - diastolic blood pressure})$$

The values of systolic and diastolic blood pressure were taken from the results of 24-h ABPM. Mean daytime values of systolic and diastolic blood pressure were used (Table 1).

**Table 1:** Mean values of diastolic and systolic blood pressure analyzed in ambulatory blood pressure monitoring

Parameters	Study group (*N* = 56)	Control group (*n* = 15)	P
24 h SBP (mmHg) 24 h DBP (mmHg)	131.12 ± 8.72 72.06 ± 7.24	118.00 ± 6.74 66.09 ± 5.45	0.01 0.01
SDS of 24 h SBP SDS of 24 h DBP	3.36 ± 1.83 1.17 ± 1.84	0.59 ± 1.28 −0.31 ± 1.38	0.01 0.01
D SBP (mmHg) D DBP (mmHg)	134.62 ± 8.69 74.79 ± 6.14	119.45 ± 6.77 68.45 ± 5.47	0.01 0.01
N SBP (mmHg) N DBP (mmHg)	121.24 ± 10.83 63.76 ± 10.52	114.09 ± 8.96 60.36 ± 3.98	0.01 Ns
MAP (mmHg)	94.98 ± 5.29	85.45 ± 4.26	0.01
SDS of MAP	1.0 ± 0.80	0.75 ± 0.69	0.01

24 h SBP—15 h mean values of systolic blood pressure; 24 h DBP—24 h mean values of diastolic blood pressure; D SBP—day mean values of systolic blood pressure; D DBP—day mean values of diastolic blood pressure; N SBP—night mean values of systolic blood pressure; N DBP—night mean values of diastolic blood pressure; MAP—mean arterial pressure

## Statistical analysis

The Shapiro–Wilk test was used to assess the data. The homogeneity of variance of each variable was calculated with Levene’s test. The non-parametric Mann–Whitney U-test was applied in the analyses of data with non-normal distribution. Student’s t-test was employed in the analyses of variables with normal distribution. Spearman’s correlation rank test was performed to analyze the correlation of parameters with non-normal distribution. Pearson’s test was employed to test the correlation of normal distribution variables. The statistical significance level was set at *p* < 0.05. Statistical analyses were conducted using Statistica 13.3 (TIBCO Software Inc.).

## Author contributions

All authors contributed to the study conception and design. Material preparation, data collection and analysis were performed by Katarzyna Maćkowiak-Lewandowicz, Anna Rzeszotarska, Marta Pawlak, Anna Gotz-Więckowska, Danuta Ostalska-Nowicka, Jacek Zachwieja. The first draft of the manuscript was written by Katarzyna Maćkowiak-Lewandowicz and all authors commented on previous versions of the manuscript. All authors read and approved the final manuscript.

## Results

Clinical data, including sex, height, height-SDS, creatinine, glucose, lipid profile, and BMI-SDS, were explicitly included (Table 1). There was a difference between the study and control groups in mean height values and SDS of height and BMI.

### Nephrological results

Children and adolescents with hypertension had decreased values of GFR (90.31 ± 13.00 ml/min/1.73 m^2^), estimated by the Filler equation compared to subjects with optimal values of blood pressure (99.00 ± 9.27 ml/min/1.73 m^2^). GFR, estimated by the Schwartz formula did not show any differences between the study and the control group. The concentration of cystatin C was increased in patients with hypertension. In both groups concentrations of sodium in urine were within normal limits. Microalbuminuria and LVMI did not differ significantly between the study and control groups (Table 2).

**Table 2:** The data of the study and control group (mean values and standard deviation)

Parameters	Study group (*n* = 56)	Control group (*n* = 15)	P
Age (yr)	13.86 ± 2.56	12.78 ± 2.96	Ns
Sex (% of girls/boys)	41/59	47/53	
Height (cm) ± SD	157.88 ± 19.48	168.6 ± 12.34	0.046
SDS of height	0.98 ± 1.67	1.36 ± 1.95	**0.04**
BMI (kg/m^2^) ± SD	25.67 ± 6.43	26.79 ± 8.37	Ns
SDS of BMI	3.77 ± 4.34	1.63 ± 2.56	**0.01**
MAP (mmHg)	94.82 ± 5.28	85.45 ± 4.25	**0.01**
Glucose (mg/dl)	90.16 ± 13.02	88.86 ± 7.47	Ns
Total cholesterol (mg/dl)	165.11 ± 29.05	162.60 ± 34.65	Ns
HDL (mg/dl)	50.95 ± 10.22	51.53 ± 9.08	Ns
LDL (mg/dl)	98.83 ± 26.04	95.93 ± 33.72	Ns
Triglycerides (mg/dl)	88.85 ± 44.52	75.40 ± 20.68	Ns
Creatinine (mg/dl)	0.62 ± 0.17	0.71 ± 0.16	Ns
Cystatin C (mg/L)	1.03 ± 0.14	0.93 ± 0.07	**0.01**
GFR F (ml/min/1.73 m^2^)	90.31 ± 13.00	99.00 ± 9.27	**0.03**
GFR S (ml/min/1.73 m^2^)	109.47 ± 18.96	101.68 ± 16.51	Ns
Microalbuminuria (mg/d)	12.85 ± 12.67	15.93 ± 16.27	Ns
24 h urinary Na (mg/d)	2103 ± 100.23	2074 ± 101.34	Ns
LVMI (g/m^2.7^)	34.83 ± 7.26	31.43 ± 7.26	Ns
SCP RE I (%)	44.46 ± 4.16	41.32 ± 4.90	0.047
SCP RE S (%)	46.11 ± 2.84	43.62 ± 5.03	Ns
SCP RE N (%)	44.68 ± 2.82	42.63 ± 3.52	**0.03**
SCP RE T (%)	45.49 ± 2.42	44.40 ± 4.29	Ns
SCP LE I (%)	44.91 ± 4.00	42.77 ± 3.42	0.046
SCP LE S (%)	45.90 ± 3.1	42.23 ± 4.2	**0.01**
SCP LE N (%)	45.68 ± 3.1	42.35 ± 4.91	**0.01**
SCP LE T (%)	45.95 ± 2.86	41.92 ± 6.49	**0.01**

P—confidence level, Ns—not significant, SD/SDS—standard deviation/standard deviation score, GFR F—glomerular filtration rate estimated by Filler equation, GFR S—glomerular filtration rate estimated by Schwartz equation, LVMI—left ventricular mass index, SCP RE (%)—right eye vessel density obtained by optical coherence tomography angiography (OCT-A) at the superficial capillary plexus (SCP) presented as the percentage of pixels occupied by blood flow, SCP LE (%)—left eye vessel density obtained by optical coherence tomography angiography (OCT-A) at the superficial capillary plexus (SCP) presented as the percentage of pixels occupied by blood flow, I—inferior quadrant, S—superior, N—nasal, T—temporal

### Ophthalmological results

Dilated fundus examination revealed stage I hypertensive retinopathy, characterized by segmental retinal arteriolar narrowing, in 7 patients (12.5%).

The data revealed significant differences in vessel density of the SCP in both eyes (in the vessel density in the inferior and nasal quadrants of the right eye and in the inferior, superior, nasal, and temporal quadrants of the left eye) between the study and control groups (Table 2). The study showed significant differences in vessel density in specific quadrants: in the inferior and nasal quadrants of the right eye, in the superior and nasal quadrants of the left eye for primary hypertension, and in multiple quadrants (inferior, superior, nasal, and temporal) in both eyes for secondary hypertension with GFR < 90 ml/min/1.73 m^2^ (Tables 3, 4). There were no differences between the study and the control groups in RNFL or choroidal thickness results in both eyes (Table 5). However, the RNFL analysis revealed that patients with hypertensive fundoscopic changes had significantly thinner RNFL in the inferior quadrant of the right eye compared to other subjects in the study group (*p* = 0.001) (Table 6). There was no statistically significant correlation between vessel density in the SCP and sex (Table I and II in supplementary material).

**Table 3:** Concentrations of kidney markers and parameters of OCT-A analyzed in group with primary hypertension and control group

Parameters	Primary hypertension *n* = 24	Control group *n* = 15	P
Creatinine (mg/dl)	0.62 ± 0.17	0.71 ± 0.16	Ns
Cystatin C (mg/L)	1.02 ± 0.12	0.93 ± 0.07	0.02
GFR F (ml/min/1.73 m^2^)	91.27 ± 11.25	99.00 ± 9.27	0.04
GFR S (ml/min/1.73 m^2^)	113.23 ± 20.34	101.68 ± 16.51	0.04
SCP RE I (%)	45.49 ± 5.24	41.32 ± 4.9	0.04
SCP RE S (%)	45.54 ± 4.47	43.62 ± 5.03	Ns
SCP RE N (%)	45.53 ± 3.69	42.63 ± 3.52	0.04
SCP RE T (%)	45.52 ± 3.23	44.40 ± 4.29	Ns
SCP LE I (%)	43.99 ± 4.32	42.77 ± 3.42	Ns
SCP LE S (%)	45.36 ± 3.96	42.23 ± 4.2	0.04
SCP LE N (%)	46.14 ± 4.05	42.35 ± 4.91	0.03
SCP LE T (%)	42.29 ± 3.67	41.92 ± 6.49	Ns

P—confidence level, Ns—not significant, SCP RE (%)—right eye vessel density obtained by optical coherence tomography angiography (OCT-A) at the superficial capillary plexus (SCP) presented as the percentage of pixels occupied by blood flow, SCP LE (%)—left eye vessel density obtained by optical coherence tomography angiography (OCT-A) at the superficial capillary plexus (SCP) presented as the percentage of pixels occupied by blood flow, I—inferior quadrant, S—superior, N—nasal, T—temporal

**Table 4:** Parameters of OCT-A in group with secondary hypertension, GFR < 90 ml/min/1.73 m^2^ and in control group

Parameters	Group with GFR < 90 ml/min/1.73m^2^ *n* = 12	Control group *n* = 15	P
SCP RE I (%)	45.47 ± 2.92	41.32 ± 4.9	0.03
SCP RE S (%)	44.34 ± 3.63	43.62 ± 5.03	Ns
SCP RE N (%)	45.14 ± 1.76	42.63 ± 3.52	0.04
SCP RE T (%)	45.66 ± 1.61	44.40 ± 4.29	Ns
SCP LE I (%)	45.15 ± 4.83	42.77 ± 3.42	0.03
SCP LE S (%)	44.3 ± 3.31	42.23 ± 4.2	0.04
SCP LE N (%)	45.14 ± 4.43	42.35 ± 4.91	0.04
SCP LE T (%)	47.39 ± 3.38	41.92 ± 6.49	0.03

P—confidence level, Ns—not significant, SCP RE (%)—right eye vessel density obtained by optical coherence tomography angiography (OCT-A) at the superficial capillary plexus (SCP) presented as the percentage of pixels occupied by blood flow, SCP LE (%)—left eye vessel density obtained by optical coherence tomography angiography (OCT-A) at the superficial capillary plexus (SCP) presented as the percentage of pixels occupied by blood flow, I—inferior quadrant, S—superior, N -nasal, T—temporal

**Table 5:** Retinal nerve fiber layer parameters (RNFL) and choroidal thickness in different quadrants of both eyes in study and control group

Parameters [µm]	Study group (*n* = 56)	Control group (*n* = 15)	P
RNFL total thickness RE	107.28 ± 28.70	103.067 ± 12.51	Ns
RNFL I RE	136.34 ± 43.29	135.33 ± 20.14	Ns
RNFL S RE	134.54 ± 42.43	123.00 ± 14.67	Ns
RNFL N RE	79.66 ± 20.33	75.53 ± 15.11	Ns
RNFL T RE	77.92 ± 19.13	78.47 ± 14.69	Ns
RNFL total thickness LE	109.52 ± 33.22	109.33 ± 25.68	Ns
RNFL I LE	140.18 ± 42.48	134.47 ± 19.96	Ns
RNFL S RE	138.96 ± 42.76	130.27 ± 15.73	Ns
RNFL N RE	82.26 ± 36.39	75.47 ± 11.21	Ns
RNFL T RE	75.78 ± 20.19	72.93 ± 11.62	Ns
Choroidal thickness subfoveal region I RE	336.26 ± 83.35	341.58 ± 50.93	Ns
Choroidal thickness N 500 um RE	322.13 ± 89.59	328.50 ± 44.02	Ns
Choroidal thickness N 1000 um RE	311.84 ± 86.52	311.42 ± 40.09	Ns
Choroidal thickness N 1500 um RE	288.68 ± 89.32	270.50 ± 40.31	Ns
Choroidal thickness T 500 um RE	326.76 ± 84.51	339.50 ± 58.23	Ns
Choroidal thickness T 1000 um RE	325.05 ± 78.97	328.25 ± 61.76	Ns
Choroidal thickness T 1500 um RE	317.57 ± 88.88	316.58 ± 64.04	Ns
Choroidal thickness subfoveal region I LE	323. 17 ± 97.22	331.67 ± 49.65	Ns
Choroidal thickness N 500 um LE	312.39 ± 94.05	317.17 ± 51.19	Ns
Choroidal thickness N 1000 um LE	295.95 ± 91.57	291.67 ± 56.83	Ns
Choroidal thickness N 1500 um LE	265.41 ± 86.74	258.58 ± 54.04	Ns
Choroidal thickness T 500 um LE	326.00 ± 95.23	339.00 ± 51.94	Ns
Choroidal thickness T 1000 um LE	322.51 ± 89.03	327.75 ± 51.62	Ns
Choroidal thickness T 1500 um LE	320.48 ± 83.22	322.33 ± 57.14	Ns

P—confidence level, Ns—not significant, RE—right eye; LE—left eye; I—inferior quadrant; S—superior quadrant; N—nasal quadrant; T—temporal quadrant; N 500 um—choroidal thickness at 500 um nasal to the fovea; T 500 um—choroidal thickness at 500 um temporal to the fovea

**Table 6:** Retinal nerve fiber layer (RNFL) parameters in group with hypertensive fundoscopic changes and study group without changes

Parameters [µm]	Group with funduscopic changes (*n* = 7)	Rest of the study group (*n* = 49)	P
RNFL total thickness RE	98.42 ± 22.86	109.14 ± 28.14	Ns
RNFL I RE	120.71 ± 33.28	136.63 ± 41.68	< 0.05
RNFL S RE	76.28 ± 17.25	81.53 ± 20.85	Ns
RNFL N RE	118.14 ± 25.13	136.63 ± 41.68	Ns
RNFL T RE	68.28 ± 22.13	79.40 ± 18.04	Ns
RNFL total thickness LE	104.86 ± 9.37	110.18 ± 33.61	Ns
RNFL I LE	135.28 ± 20.89	141.33 ± 43.15	Ns
RNFL S RE	81 ± 4.16	82.53 81 ± 36.85	Ns
RNFL N RE	132.85 ± 12.53	139.37 ± 43.31	Ns
RNFL T RE	70.42 ± 6.54	76.49 ± 20.67	Ns

P—confidence level, Ns—not significant; RE—right eye; LE—left eye; I—inferior quadrant; S—superior quadrant; N—nasal quadrant; T—temporal quadrant

The results of kidney markers and parameters of OCT-A in the group with primary hypertension and secondary hypertension in comparison to the control group are presented in supplementary material (Table III and IV).

### Correlations

There was a significant, yet weak correlation between MAP and vessel density in the temporal quadrant of the left eye (*p* = 0.03, R = 0.356), and caution in interpreting this asymmetry between eyes was noted. Only values of mean 24-h systolic blood pressure and mean daily systolic blood pressure correlated with values analyzed in OCT-A in the left and right eye in the superior quadrant, but correlation levels were weak (r = 0.3249; r = 0.3270). The rest of the analyzed ophthalmologic parameters did not correlate with values from ABPM. There was no significant correlation between LVMI and analyzed ophthalmological parameters.

## Discussion

Hypertensive retinopathy is only one possible ophthalmological sequela caused by increased blood pressure. However, it is completely asymptomatic at stages 1 and 2 under the Keith, Wagener, and Barker classification. The physician can observe hypertensive alterations in the microvasculature in vivo during fundoscopy, which may reflect patients’ general health and provide prognostic information for cardiovascular risk stratification. While signs of hypertensive retinopathy can be detected in 6–15% of the population aged ≥ 40 years, this number is thought to be significantly underestimated [20]. In our study, stage 1 retinopathy under the Keith, Wagener, and Barker classification was detected in 12.5% of patients with hypertension. Similar results were presented in the study by Mackie et al., where the authors found symptoms of hypertensive retinopathy in only 8.6% of evaluated children and all retinal changes were described as “mild abnormalities” [21]. It should also be emphasized that the evaluation of hypertensive retinopathy relies on subjective fundoscopic findings, which, according to some authors, may be subject to interobserver variability [22]. Therefore, more objective and repetitive diagnostic methods should be employed.

Several studies on adult patients with systemic hypertension have evaluated the RNFL in the peripapillary region. Özkan et al. found that peripapillary RNFL parameters were significantly lower among adult patients with systemic arterial hypertension than among healthy subjects [7]. In addition, a longitudinal, four-year observational study by Kim et al. showed a significantly greater decrease in peripapillary RNFL among patients with hypertension than among healthy individuals [23]. Our study did not reveal significant differences in the peripapillary RNFL between the study and the control group. RNFL analysis demonstrated that patients with hypertensive fundoscopic changes had significantly thinner RNFL only in the inferior quadrant of the right eye compared to other subjects in the study group. Patients in our study had been newly diagnosed with systemic hypertension, and perhaps only a prolonged increase in blood pressure affects peripapillary RNFL. Therefore, longitudinal observational studies are required to confirm this theory.

Elevated blood pressure might affect the choroid, a highly vascularized tissue that provides blood supply to the outer two-thirds of the retina. While several methods exist to image the choroid in vivo, spectral domain OCT (SD-OCT) or swept-source OCT (SS-OCT) supports precise measurements of choroidal thickness since it markedly delineates the choroid-sclera interface [24]. Mittal et al. reported that choroidal thickness was thinner in patients with systemic hypertension and found a significant but weak negative correlation between systolic blood pressure and hypertension duration [25]. Interestingly, Uzum et al. reported similar observations concerning decreased choroidal thickness in young adults (mean age: 23.8 ± 2.8 years) diagnosed with systemic hypertension. Unlike these previous studies, we did not find choroidal thickness to differ significantly between the study and control groups. One possible reason might be the longer disease duration – even in the study by Uzum et al., the mean systemic hypertension diagnosis duration was 3.4 ± 1.4 years [26]. In our study group all patients were newly diagnosed.

The results of a meta-analysis by Chua et al. suggested that OCT-A can provide information about pre-clinical microvascular changes secondary to systemic hypertension. All studies analyzed in this meta-analysis focused on middle-aged and older patients with newly diagnosed or already treated systemic hypertension. Furthermore, other types of OCTA machines (AngioVue, PLEX Elite 9000, and Cirrus AngioPlex 5000) were used [27]. Therefore, the findings of this meta-analysis cannot be compared with ours. However, our findings may be considered thought-provoking. Specifically, the vessel density in the retinal SCP might be decreased in patients with hypertension due to functional non-perfusion or structural absence of capillaries. Our results show the opposite trend – subjects in the control group tended to have significantly lower vessel densities at the retinal SCP than those in the study group. It should be mentioned that the control group in our study consisted of patients who were admitted to the hospital as hypertension suspects. Eventually, after thorough medical evaluation, this diagnosis was excluded. We cannot exclude the possibility that these patients may present with a diagnosis of hypertension in the future. Therefore, in these patients, the follow-up should be considered as well. Moreover, the participants in the control group had increased body mass index (BMI). As presented in the study by Ding et al. increased BMI can be associated with increased macular vessel density in the SCP [28]. This explains how the observed higher BMI-SDS in the hypertension group could potentially contribute to the higher SCP values found in the control group. Several studies have examined the impact of obesity and increased BMI on the retinochoroidal vasculature. Some found increased vessel density in the SCP, while others suggested decreased vessel density but in the deep capillary plexus (DCP) [29, 30]. Possibly, other comorbidities such as obesity affect retinal vasculature earlier than hypertension. However, it should be emphasized that we found statistically significant differences in SDS of BMI between the study and the control group and no differences in BMI values. Definitely, further research and follow-up might clarify these findings.

Assessing microvascular alterations in kidneys is challenging. Evaluation of carotid intima-media thickness and pulse wave velocity, which is an indirect marker of arterial stiffness is not a routine examination in children and adolescents with hypertension [31]. Microvascular remodeling decreases perfusion, leading to tubular ischemia. The first changes in microcirculation likely start not only in the retina but also in the proximal tubules, where cystatin C, after being filtered without restriction by the glomeruli, is entirely reabsorbed by the proximal tubules, where it is almost entirely catabolized [32, 33]. Cystatin C is a functional marker of early kidney damage, an indicator of filtration and might be a non-specific indicator of renal microcirculatory alterations. The impaired autoregulation in hypertensive kidneys exacerbates glomerular filtration. Dysfunction of the highly metabolically active nephron segments, such as proximal tubules, seems to be the underlying cause of kidney injury [34].

Our study revealed significant differences between the study and control groups in cystatin C and GFR concentrations assessed by the Filler equation. The increased concentrations of cystatin C observed in patients with hypertension can be the effect of kidney function, especially in the group with secondary nephrological hypertension, but also an indirect indicator of proximal tubule changes, as an effect of microvascular remodeling. Additionally, untreated hypertension results in proteinuria. Our study found no significant differences in the presence of microalbuminuria between the study and control groups.

There was a weak but statistically significant correlation between MAP values and vessel density in the SCP; however only in the left eye. Drawing proper conclusions considering these results could give broad implications at this moment, but the larger number of patients in both groups, as well as follow-up, may be helpful to resolve this issue.

Our study had several significant limitations. Firstly, the number of patients in the study and control groups differed. The control group consisted of patients, who at the beginning were admitted to the hospital for clinical evaluation, as they were suspected of having hypertension. Final evaluation excluded any diagnosis of hypertension or other chronic conditions. They did not meet the criteria for white coat hypertension. They are "hospital-based healthy controls" rather than community-recruited healthy subjects. The study group is heterogeneous and includes patients both with primary and secondary hypertension and subgroups of secondary hypertension, especially kidney etiology with hypofiltration, which could have an influence on concentrations of cystatin C. The study and control groups differed in mean height values and SDS of height and BMI. Moreover, kidney microvascular changes were indirectly assessed through markers like cystatin C and eGFR calculated with the Filler equation. Cystatin C is a functional marker of early kidney damage, an indicator of filtration and might be an indirect marker of renal microcirculatory alterations rather than a proximal tubule-specific biomarker. We did not analyse additional tubular markers such as NGAL or KIM-1 in the presented study. Further research with additional proximal tubule markers needs to be conducted to verify the results and hypothesis.

In ophthalmological testing, OCT was performed only once, with no follow-up. The OCT-A mode in DRI-Triton Topcon does not permit a quantitative analysis of the choriocapillaris or DCP of the macula. Also, due to methodological limitations, we did not assess the foveal avascular zone (FAZ). Moreover, OCT and OCT-A parameters may be affected by sex, age, race, or axial length. The DRI Triton Topcon, Japan, which we used in our study, includes a normative database for statistical comparison of the thickness maps and parameters. Unfortunately, the Topcon Triton Swept Source OCT system (like other OCT systems) does not have a dedicated normative database for the paediatric population. Paediatric OCT databases are available only in scientific literature, and they comprise RNFL data from various OCT systems [36]. The same applies to OCT-A parameters, where Heidelberg OCT software was employed [37]. We should also mention that the OCT-A parameters can be affected by the signal strength index (SSI) [35]. Higher SSI indicates better image quality. Although we tried to obtain the best accuracy of all scans in all participants and assess the best accuracy, later we did not consider SSI in the statistical analysis. Considering these inconveniences, we decided to eventually compare the results from the study group with our control group.

The number of publications referring to hypertension among children and its impact on the kidneys or retina is limited. Therefore, it should be emphasized that our paper represents valuable material regarding this matter.

Early detection of microvasculature dysfunction within the retina or kidney dysfunction may play a significant role in prophylaxis and prevention of irreversible organ damage. Creating a proper monitoring system could be a good solution; however, our knowledge regarding this area is still limited.

## Conclusions

Pediatric patients with newly diagnosed hypertension have increased concentrations of cystatin C and hypofiltration estimated by the Filler equation.

Although OCT-A can be a tool to diagnose early complications of hypertension in adults, further studies are necessary to better understand the timeline of retinal microvascular changes in hypertension in children, as well as the impact of other comorbidities in this process. However, as a non-invasive technique, it might be considered an additional diagnostic tool in this group of patients.

## Supplementary Information

Below is the link to the electronic supplementary material.

Graphical abstract (PPTX 120 KB)

ESM 2 (DOCX 23.1 KB)

## Data Availability

The data of study is availability for Journal.
