# Supplementary material for: Retinal vascular density in children with hypertension
Source: Pediatr Nephrol. 2026 Jan 8;41(5):1415–24. doi: 10.1007/s00467-025-07076-7 (PMC13009065; doi:10.1007/s00467-025-07076-7)
Supplement: Supplementary file 1 — Graphical abstract (PPTX 120 KB) [file 467_2025_7076_MOESM1_ESM.pptx]

## Slide 1
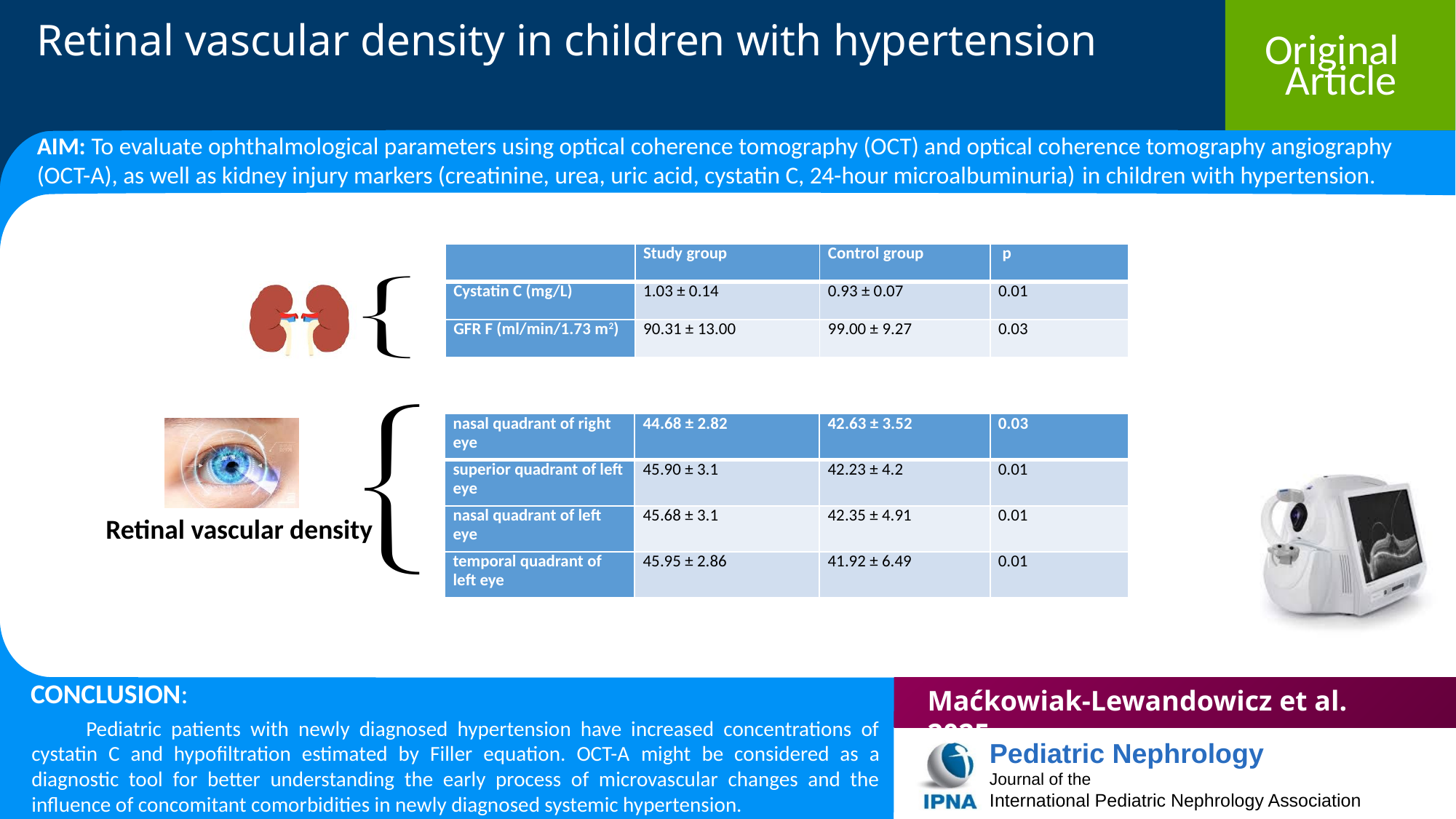

Retinal vascular density in children with hypertension
AIM: To evaluate ophthalmological parameters using optical coherence tomography (OCT) and optical coherence tomography angiography (OCT-A), as well as kidney injury markers (creatinine, urea, uric acid, cystatin C, 24-hour microalbuminuria) in children with hypertension.
| | Study group | Control group | p |
| --- | --- | --- | --- |
| Cystatin C (mg/L) | 1.03 ± 0.14 | 0.93 ± 0.07 | 0.01 |
| GFR F (ml/min/1.73 m2) | 90.31 ± 13.00 | 99.00 ± 9.27 | 0.03 |
| nasal quadrant of right eye | 44.68 ± 2.82 | 42.63 ± 3.52 | 0.03 |
| --- | --- | --- | --- |
| superior quadrant of left eye | 45.90 ± 3.1 | 42.23 ± 4.2 | 0.01 |
| nasal quadrant of left eye | 45.68 ± 3.1 | 42.35 ± 4.91 | 0.01 |
| temporal quadrant of left eye | 45.95 ± 2.86 | 41.92 ± 6.49 | 0.01 |
Retinal vascular density
CONCLUSION:
Maćkowiak-Lewandowicz et al. 2025
Pediatric patients with newly diagnosed hypertension have increased concentrations of cystatin C and hypofiltration estimated by Filler equation. OCT-A might be considered as a diagnostic tool for better understanding the early process of microvascular changes and the influence of concomitant comorbidities in newly diagnosed systemic hypertension.
