# Supplementary material for: Retinal vascular density in children with hypertension
Source: Pediatr Nephrol. 2026 Jan 8;41(5):1415–24. doi: 10.1007/s00467-025-07076-7 (PMC13009065; doi:10.1007/s00467-025-07076-7)
Supplement: Supplementary file 2 — (DOCX 23.1 KB) [file 467_2025_7076_MOESM2_ESM.docx]

Table I. Analysis of angio-OCT parameters between boys and girls in study group

| **variables** | **Boys n=33** | **Girls n=23** | **p** |
| --- | --- | --- | --- |
| SCP RE I (%) | 44.27 ± 3.85 | 41.70 ± 5.47 | >0.05 |
| SCP RE S (%) | 44.69 ± 3.69 | 44.44 ± 4.72 | >0.05 |
| SCP RE N (%) | 44.79 ± 3.15 | 43.10 ± 4.05 | >0.05 |
| SCP RE T (%) | 45.89 ± 3.39 | 44.34 ± 2.09 | >0.05 |
| SCP LE I (%) | 44.17 ± 4.03 | 42.33 ± 4.73 | >0.05 |
| SCP LE S (%) | 43.31 ± 3.48 | 44.05 ± 3.37 | >0.05 |
| SCP LE N (%) | 44.79 ± 2.73 | 44.06 ± 4.39 | >0.05 |
| SCP LE T (%) | 45.42 ± 2.95 | 43.93 ± 3.72 | >0.05 |

P - confidence level, SCP RE (%) – right eye vessel density obtained by optical coherence tomography angiography (OCT-A) at the superficial capillary plexus (SCP) presented as the percentage of pixels occupied by blood flow, SCP LE (%) – left eye vessel density obtained by optical coherence tomography angiography (OCT-A) at the superficial capillary plexus (SCP) presented as the percentage of pixels occupied by blood flow, I- inferior quadrant, S-superior, N-nasal, T-temporal

Table II. Analysis of angio-OCT parameters between boys and girls in control group

| **variables** | **Boys n=8** | **Girls n=7** | **p** |
| --- | --- | --- | --- |
| SCP RE I (%) | 42.55 ± 4.72 | 41.84 ± 4.43 | >0.05 |
| SCP RE S (%) | 43.3 ± 4.46 | 42.9 ± 5.42 | >0.05 |
| SCP RE N (%) | 43.73 ± 3.05 | 42.0 ± 3.87 | >0.05 |
| SCP RE T (%) | 44.89 ± 3.19 | 43.92 ± 5.35 | >0.05 |
| SCP LE I (%) | 44.20 ± 1.93 | 41.9 ± 4.42 | >0.05 |
| SCP LE S (%) | 44.33 ± 2.11 | 41.5 ± 5.75 | >0.05 |
| SCP LE N (%) | 45.61 ± 1.55 | 41.25 ± 6.74 | >0.05 |
| SCP LE T (%) | 45.52 ± 2.18 | 40.87 ± 9.35 | >0.05 |

P - confidence level, SCP RE (%) – right eye vessel density obtained by optical coherence tomography angiography (OCT-A) at the superficial capillary plexus (SCP) presented as the percentage of pixels occupied by blood flow, SCP LE (%) – left eye vessel density obtained by optical coherence tomography angiography (OCT-A) at the superficial capillary plexus (SCP) presented as the percentage of pixels occupied by blood flow, I- inferior quadrant, S-superior, N-nasal, T-temporal

Table III. Concentrations of kidney markers and parameters of angio-OCT analyzed in group with primary hypertension and control group

| **Parameters** | **Primary hypertension**  **n= 24** | **Control group**  **n = 15** | **P** |
| --- | --- | --- | --- |
| Creatinine (mg/dl) | 0.62 ± 0.17 | 0.71 ± 0.16 | ns |
| Cystatin C (mg/L) | 1.02 ± 0.12 | 0.93 ± 0.07 | 0.02 |
| GFR F (ml/min/1.73m^2^) | 91.27 ± 11.25 | 99.00 ± 9.27 | 0.04 |
| GFR S (ml/min/1.73m^2^) | 113.23 ± 20.34 | 101.68 ± 16.51 | 0.04 |
| SCP RE I (%) | 45.49 ± 5.24 | 41.32 ± 4.9 | 0.04 |
| SCP RE S (%) | 45.54 ± 4.47 | 43.62 ± 5.03 | ns |
| SCP RE N (%) | 45.53 ± 3.69 | 42.63 ± 3.52 | 0.04 |
| SCP RE T (%) | 45.52 ± 3.23 | 44.40 ± 4.29 | ns |
| SCP LE I (%) | 43.99 ± 4.32 | 42.77 ± 3.42 | ns |
| SCP LE S (%) | 45.36 ± 3.96 | 42.23 ± 4.2 | 0.04 |
| SCP LE N (%) | 46.14 ± 4.05 | 42.35 ± 4.91 | 0.03 |
| SCP LE T (%) | 42.29 ± 3.67 | 41.92 ± 6.49 | ns |

p - confidence level, ns -not significant, SCP RE (%) – right eye vessel density obtained by optical coherence tomography angiography (OCT-A) at the superficial capillary plexus (SCP) presented as the percentage of pixels occupied by blood flow, SCP LE (%) – left eye vessel density obtained by optical coherence tomography angiography (OCT-A) at the superficial capillary plexus (SCP) presented as the percentage of pixels occupied by blood flow, I- inferior quadrant, S-superior, N-nasal, T-temporal

Table IV. Concentrations of kidney markers and parameters of angio-OCT in group with secondary hypertension and in control group

| **Parameters** | **Secondary hypertension**  **n = 32** | **Control group**  **n = 15** | **P** |
| --- | --- | --- | --- |
| Creatinine (mg/dl) | 0.72 ± 0.56 | 0.71 ± 0.16 | ns |
| Cystatin C (mg/L) | 1.05 ± 2.29 | 0.93 ± 0.07 | 0.03 |
| GFR F (ml/min/1.73m^2^) | 84.78 ± 28 | 99.00 ± 9.27 | 0.02 |
| GFR S (ml/min/1.73m^2^) | 102.77 ± 23.69 | 101.68 ± 16.51 | ns |
| SCP RE I (%) | 43.48 ± 3.6 | 41.32 ± 4.9 | 0.03 |
| SCP RE S (%) | 44.17 ± 3.00 | 43.62 ± 5.03 | ns |
| SCP RE N (%) | 43.48 ± 3.13 | 42.63 ± 3.52 | ns |
| SCP RE T (%) | 44.93 ± 2.79 | 44.40 ± 4.29 | ns |
| SCP LE I (%) | 43.39 ± 3.89 | 42.77 ± 3.42 | ns |
| SCP LE S (%) | 45.03 ± 2.89 | 42.23 ± 4.2 | 0.04 |
| SCP LE N (%) | 44.15 ± 2.89 | 42.35 ± 4.91 | 0.04 |
| SCP LE T (%) | 44.49 ± 2.95 | 41.92 ± 6.49 | 0.04 |

p - confidence level, ns -not significant, SCP RE (%) – right eye vessel density obtained by optical coherence tomography angiography (OCT-A) at the superficial capillary plexus (SCP) presented as the percentage of pixels occupied by blood flow, SCP LE (%) – left eye vessel density obtained by optical coherence tomography angiography (OCT-A) at the superficial capillary plexus (SCP) presented as the percentage of pixels occupied by blood flow, I- inferior quadrant, S-superior, N-nasal, T-temporal
